# Innovation diffusion: how homogenous networks influence the uptake of community-based injectable contraceptives

**DOI:** 10.1186/s12889-019-7819-5

**Published:** 2019-11-14

**Authors:** Oluwaseun Akinyemi, Bronwyn Harris, Mary Kawonga

**Affiliations:** 1 Department of Health Policy and Management, College of Medicine, |University of Ibadan, Ibadan, Nigeria; 2 Department of Community Health, School of Public Health, Faculty of Health Sciences, University of the Witwatersrand, Johannesburg, South Africa; 3 Centre for Health Policy, School of Public Health, Faculty of Health Sciences, University of the Witwatersrand, Johannesburg, South Africa; 4 Division of Health Sciences, Warwick Medical School, University of Warwick, Coventry, UK

**Keywords:** Ego social network analysis, Personal networks, Egocentric networks, Community-based distribution of injectable contraceptives, Homophily, Density, Innovation diffusion, Policy analysis, Contraceptive policy, Nigeria

## Abstract

**Background:**

Studies have shown that social networks influence health behaviors, including the adoption of health innovations. This study explored the potential for early adopters of community health worker-delivered injectable contraceptives (CHWDIC) to influence the uptake of this innovation by women in their social networks.

**Methods:**

This Social Network Analysis (SNA) study was conducted in Gombe, Nigeria. Twenty women who were early adopters of the CHWDIC were recruited. Each participant (ego) listed ten women of reproductive age (alters) with whom they related. An interviewer-administered questionnaire was used to collect from each ego, data about the nature of her relationship with each alter (ego-alter relationship), whether she talked about CHWDIC with each alter, and whether her listed alters talked to each other about CHWDIC (alter-alter relationship). Data were also collected on age, marital status and education level for each ego and alter. Data were analyzed with UCINET social network analysis software. Variables of interest include homophilia (similarity), density (number of ties as a proportion of possible ties), degree (popularity) and betweeness (frequency of connecting actor pairs who otherwise might not communicate).

**Results:**

There were 20 egos and 200 alters. Between two thirds (alters) and three quarters (egos) of the women were 30 years or older. All of the egos and 196 (98%) of alters were married. Most of the networks had similar (homophilic) actors according to certain sociodemographic characteristics - ethnicity, age, education and type of marriage. More than 90% of the networks had density greater than 50%, suggesting high cohesion in most networks. The majority of actors in these networks used injectable contraceptives. In some of the networks, few actors with the highest prominence (betweeness centrality) were not users of injectable contraceptives.

**Conclusion:**

The study illustrates the application and feasibility of ego SNA in identifying champions and opinion leaders among women of reproductive age group. It also shows the influence of social networks on the diffusion of community-based injectable contraceptives, and how homophilic and dense networks may have positive health externality. The interrelatedness of network members’ decision to adopt a health innovation was also demonstrated by the findings of this study.

## Introduction

Studies have shown, particularly in developing countries, that for health innovations to achieve their aims, there is a need for scale up [1, 2]. Scale up entails making the innovation available to a larger population and/or to new locations through better financing, and provision of material and human resources, as well as an improved public health delivery system [1, 2]. Studies show that social networks can facilitate both positive and negative behaviors [3, 4] – either through word of mouth or virtual networks via social media [5]. Also, social networks can support uptake of health innovation, and thus facilitate scale up [3, 6].

Although researchers [4, 6, 7] have demonstrated that social networks can support the uptake of health innovation, there is limited understanding or guidance on the role of social networks in the scale up of community-based injectable contraceptives- an innovation adopted in Nigeria. Uptake of this intervention is particularly important in Nigeria, a country with low contraceptive prevalence rates (CPR) and unmet need for contraception [8]. CPR is 14.6% for any method (rhythm, withdrawal, traditional methods) and 9.7% for modern methods (pills, condoms, implants, intrauterine devices, injectable contraceptives) [9, 10], with a significantly lower rate in rural settings [11, 12]. Furthermore, only 3% of married women in Nigeria use an injectable contraceptive method [13]. However, the main thrust of Nigeria’s National Policy on Population was to reduce the country’s high rate of fertility (currently 5.5 births per woman) by encouraging voluntary adoption of family planning, in particular, modern contraceptive methods including injectable contraceptive methods [13]. Injectable contraceptive is the most popular method among women of reproductive age group in Nigeria [14]. Evidence from other African countries showed that injectable contraceptive was more effective in preventing pregnancies compared to other contraceptives because its effect is long-term and puts the woman in control [13, 14]. In 2010, a pilot study in Gombe State (one of the 36 states in Nigeria), tested the feasibility of community health extension workers (CHEWs) distributing injectable contraceptives to users at households and other settings outside the health facility with the aim to increase contraceptive prevalence rate [14]. Following the success of the pilot, the intervention was scaled-up in other parts of Gombe and later extended to another state, with plans to ultimately make the benefits available throughout the country [14]. Research exploring the potential of social networks in innovation uptake is sparse, especially in low- and middle-income countries (LMICs) like Nigeria [6, 15].

## Background

The community-based distribution of injectable contraceptives by community health extension workers has been implemented in pilot projects around the world, with significant improvement in contraceptive prevalence rate [16–18]. Research has shown that community-based distribution of family planning commodities improves access to and uptake of family planning methods [19]. This innovation involves the provision of injectable contraceptives to women of reproductive age group by community health extension workers in the community. It has been shown to be safe and effective in meeting couples’ unmet need for contraception in demonstration projects in Kenya, Rwanda, Ethiopia, Malawi and Uganda [20, 21].

Many health innovations have been shown to improve health [22]. These include interventions to combat AIDS, malaria, tuberculosis as well as to improve reproductive health [22–24]. Although some health innovations are effective in demonstration projects [16, 23], they might not be made available to a large part of the population, or, where available, may be inequitably distributed [14, 16]. Also, there is limited information about how to successfully disseminate, diffuse and scale up such innovations on a wider scale [16].

### Social networks and scale up of innovations

A social network consists of actors joined together through one or more ties or relations [25]. Through these ties, actors exchange information and also transmit expertise, knowledge, experience, and behaviors [3]. Social network analysis studies are used to assess various features of social networks, some of which are relevant for this study. For example, density – which is the number of ties forged within a network as a proportion of total possible ties – is a measure of cohesion amongst network actors. Degree centrality describes the position that each actor occupies in a network. In a network, some actors are central or prominent (those with the greatest number of ties to others), while others may be peripheral (those with few ties or direct connections to others). Another network feature is homophily, which is a measure of sameness or how much an actor relates with other actors with similar characteristics [26]. Furthermore, betweeness centrality measures the number of times an actor connects individuals who may otherwise not be connected in the network [27, 28]. In particular, this study seeks to find answers to the following questions: Do early adopters share information about the innovation with other women in their social circles? How tightly knit are these social networks and are there prominent actors in them? Who are the women they share with? Do women share this kind of information with those of similar characteristics? What characteristics commonly bind actors in these women’s social networks together? In which social settings do they regularly interact (opportunities for sharing)?

In order to answer the questions above, we utilized social network analysis (SNA) to explore the potential for early adopters of community-distributed injectable contraceptives to influence the uptake of the innovation by new users in their social networks. The early adopters were women of the reproductive age group who had adopted the community-based injectable contraceptives at the time of the pilot study in Gombe state. To the best of our knowledge, no study has explored the role of social networks in the diffusion of injectable contraceptives in Nigeria, especially from users’ perspectives. Thus, this study describes features (density and homophily) of the social networks of early adopters of community-distributed injectable contraceptives and explores the extent to which early adopters talked to / shared information about the innovation with women in their social networks.

In the AIDED model, Bradley and colleagues [29] conceptualized scale up as a process that entails various interrelated stages. These stages include assessing the landscape, innovating to fit potential users’ interests, developing community support, engaging with users of innovation and devolving in order to enhance innovation spread. According to Bradley et al., [16] spread of health innovations from early adopters (index users) in low-income countries often occur via their social networks. However, there is a dearth of information on the Devolve component of the AIDED model and how social networks facilitate the scale up of injectable contraceptives among user groups [30]. The Devolve component entails diffusion of innovation through the peer networks of the initial users [30]. In addition, Valente, in his review of research evidence, submitted that social networks influence how people adopt new ideas and it may enhance behavioral change, organizational efficiency as well as the diffusion and spread of innovations [3, 31]. The decision to adopt or reject a new idea is mostly based on subjective evaluations from peers and hardly on research evidence [32]. Therefore, interpersonal communication channels have been found to be more effective than mass media in the formation and sustenance of attitudes towards an innovation since the diffusion of innovations is essentially a social process entailing the exchange of ideas among people [32]. Hence the use of social networks in understanding the spread of community-based injectable contraceptives in this study. This SNA study draws from the “Devolve” component of the AIDED model. SNA is a method that enables us to study social networks and how these influence behaviors and spread of innovation [3].

## Methods

### Study setting

Nigeria is a federal state with 36 federating units/states and a federal capital territory – Abuja. Health is governed at the federal, state and local government levels. This study was conducted in Gombe State (North East). It is largely rural, with about 80% of the population engaged in Agriculture. Gombe is divided administratively into 11 Local Government Areas (LGAs) and has a population of about 2.4 million people [33] Gombe State is multi-ethnic with the Hausa/Fulani being the dominant ethnic group, while Hausa language is widely spoken in the State [34]. The state is a patriarchal, culturally conservative setting with a predominantly Muslim population [33]. It borders Borno, the epicenter of the *Boko Haram* insurgency, to the west. Gombe State has suffered sporadic attacks by insurgents in the past few years [35, 36]. About 46% of married women in the state live in polygamous unions compared with a national average of 33% [37]. Polygamy is inversely proportional to educational level and wealth quintiles. Among women aged 25–49 years, the median age of first marriage is 15.8 years in Gombe State compared to a regional median of 17.5 years, while the median age of sexual debut is 15.9 years compared to the national median age of 17.6 years [35]. According to the 2013 Nigerian Demographic and Health Survey, 82.1% of women in Gombe make independent decisions about their earnings versus 70% national average, although the majority of the women in the state (81.0%) earn less than the men [37].

The State has a total fertility rate of 7.4 and one of the lowest contraceptive prevalence rates in the country (3.5% for modern methods and 4.0% for any method) [10, 14, 33]. The condition of public sector health facilities in North East region seems much better compared to the private sector facilities using the percent distribution of live births in these sectors as a proxy (18.4% vs. 1.2% for public and private health sectors respectively) [13].

Between 2008 and 2010, the community-based access to injectable contraceptive pilot project was implemented in two LGAs of Gombe State (Funakaye and Yamaltu/Deba) by the Nigerian Ministry of Health, with support from the Association for Reproductive and Family Health (a national NGO) and FHI 360 (an international NGO) [14]. This study, which is part of broader research to explore the scale up of injectable contraceptives, was conducted in Gombe and Yemaltu/Deba LGAs. In 2014, the Association for Reproductive and Family Health led the scale up process, starting with the training of trainers and community health extension workers [38]. By 2016, large scale provision of injectable contraceptives at the community level commenced in Gombe State and Kebbi State (North West), with a plan to activate the scale up in Ebonyi State (South East Nigeria).

### Study design and sampling

This study was part of a larger research to assess the scale up of community health worker-delivered injectable contraceptives in Gombe State Nigeria. We used an ego social network analysis and study design. Ego social network, also known as personal network, comprises of a focal actor (ego) as well as other actors (alters) connected to the ego through one or more relations [27]. In ego network design, data on the ties between ego and alters as well as between alters (alter-alter relationship) are documented entirely from the ego’s perspective [27]. In this study, the egos were the early adopters of the community-based injectable contraceptives.

Twenty women of reproductive age group (egos), 10 each from Gombe and Yemaltu-Deba LGAs (Site A and Site B respectively) who had earlier participated in focus group discussions as part of the bigger study exploring the scale up of community-based distribution of injectable contraceptives in Gombe, were sampled purposively and recruited to participate in the social network analysis study. Each ego was requested to list ten women in the reproductive age group (15–49) (alters), with whom she had a social relationship (regardless of whether they used injectable contraceptives or not).

### Data collection

During the survey, each ego was asked to list the initials of 10 alters (women in their social and peer networks). We presented each ego with this list and asked her to specify the nature of the social relationship with each alter. Then we collected data on the relation of interest - communication about the community-based distributed injectable contraceptives. Each ego (early adopter) was asked to specify whether they shared the relation of interest with each alter (ego-alter communication relation) and to state the frequency of communication about community-based distributed injectable contraceptives. An interviewer-administered questionnaire (see Additional file 1) was administered to the egos to collect their sociodemographic and data on network variables, as well as those of alters. In addition, information on ego-alter relationship and discussions on community-based distribution of injectable contraceptives, including alter-alter relationship were collected in this questionnaire. Also, the egos were asked whether they think that alters share information about injectable contraceptives among themselves (alter-alter communication relation) and the likelihood that alters will recommend community-based injectable contraceptives to one another. Frequencies and proportions, as well as specific SNA variables were generated. The interviews, each taking about 1 h, were conducted by the lead author assisted by two female research assistants in September 2016.

### Data analysis

Data from the SNA questionnaires were imported from Microsoft Excel into UCINET social network software, (http://www.analytictech.com/) from where variables were generated. Twenty-one-mode matrices were developed (one per ego network). Likewise, 20 sociograms (one per ego network) were generated. Each ego network depicts ego-alter as well as alter-alter relations. Every node in the network maps represents an actor.

## Results

### Socio-demographic characteristics of egos and their alters

As shown in Table 1, about two-thirds (61.5%) and three-quarters (75.0%) of alters and egos were 30 years or older. Almost all the actors (98.0% of alters and all egos) were married, with more than half (53.7%) in monogamous relationships. Of the 220 actors, 81% were Hausas, slightly more than half (55.0%) had primary or secondary school education and 58% were housewives or informally employed.

**Table 1:** Sociodemographic characteristics of actors in the networks (*N* = 220)

Variable	Egos (*N* = 20)		Alters (*N* = 200)		Total	
	n	%	n	%	n	%
Age group						
< 30	5	25.0	77	38.5	82	37.3
≥ 30	15	75.0	123	61.5	138	62.7
Marital status						
Single	0	0	4	2.0	4	1.8
Married	20	100	196	98.0	216	98.2
Highest level of education						
No formal education	3	15.0	53	26.5	56	25.5
Primary or secondary education	12	60.0	109	54.5	121	55.0
Tertiary education	5	25.0	38	19.0	43	19.5
Occupation						
Informal	9	45.0	119	59.9	128	58.2
Formal occupations	6	30.0	46	23.0	52	23.6
Self-employed/employer	5	25.0	35	17.5	40	18.2
Ethnicity						
Hausa	16	80	161	80.5	177	80.5
Others^a^	4	20.0	39	19.5	43	19.5
Marriage type			(*N* = 196)		(*N* = 216)	
Monogamy	11	55.0	105	53.6	116	53.7
Polygamy	9	45.0	91	46.4	100	46.3
Use of contraceptives/family planning						
Yes	20	100	184	92.0	203	92.3
No	0	0	16	8.0	16	7.3
Use of CBD injectable contraceptives						
Yes	20	100	165	82.5	185	84.1
No	0	0	35	17.5	35	15.9

^a^Others include Igbo, Tangale, Tera, Waja

The networks were labeled A to T. In most of the networks – in both sites, the ego was slightly older than her alters (Table 2).

**Table 2:** Age of egos and alters by network

Network	Age of ego (years)	Age of alters (years) – mean (±SD)
SITE A (GOMBE)		
SNA-A	38.0	33.2 ± 3.7
SNA-B	40.0	34.9 ± 6.4
SNA-C	38.0	36.1 ± 6.2
SNA-D	35.0	32.4 ± 5.0
SNA-E	30.0	35.5 ± 4.8
SNA-F	37.0	36.6 ± 5.0
SNA-G	36.0	31.3 ± 2.3
SNA-H	36.0	33.0 ± 2.5
SNA-I	36.0	31.9 ± 1.9
SNA-J	34.0	32.5 ± 2.2
SITE B (YEMALTU-DEBA)		
SNA-K	35.0	30.5 ± 5.5
SNA-L	37.0	29.8 ± 5.5
SNA-M	40.0	32.0 ± 6.6
SNA-N	23.0	28.6 ± 5.5
SNA-O	25.0	24.8 ± 5.5
SNA-P	25.0	22.7 ± 2.1
SNA-Q	32.0	24.2 ± 5.8
SNA-R	30.0	26.9 ± 9.6
SNA-S	22.0	27.7 ± 9.9
SNA-T	20.0	22.9 ± 4.2

Based on egos’ reported accounts, 184 (92.0%) of alters used family planning and 165 (82.5%) were using CBD injectables at the time of the study.

### Social relationships between ego and alters

According to the egos, 54% of alters were relatives or close friends of ego, while 35% were neighbors or acquaintances of ego. Interaction through visiting with each other was the most common means of social contact between egos and their alters (58.5%), followed by communication at the market or workplace (25.5%) and at places of worship (16.0%). About 178 (89%) of the alters interacted with their ego once a month or more frequently. Ego-alter discussion on family planning was done often with almost all alters (96.0%). According to the egos, more than 90% of alters used contraceptives or a form of family planning method while more than 80% used CBD injectable contraceptives (Table 3).

**Table 3:** Alters’ relationship with ego (*N* = 200)

Variable	SITE A		SITE B		Total	
	n	%	n	%	n	%
Relationship with ego						
Relatives and close friends	36	36.0	72	72.0	108	54.0
Co-workers	15	15.0	7	7.0	22	11.0
Neighbour and acquaintance	49	49.0	21	21.0	70	35.0
Place of interaction with ego						
Mosque/church	19	19.0	13	13.0	32	16.0
Market and workplace	37	37.0	14	14.0	51	25.5
Social visits	44	44.0	73	73.0	117	58.5
Frequency of social interaction with ego						
At least once weekly	22	22.0	54	54.0	76	38.0
At least once monthly	61	61.0	41	41.0	102	51.0
At least once yearly	17	17.0	5	5.0	22	11.0

### Narrative of network composition

#### Network density and centralization

**Fig. 1:**
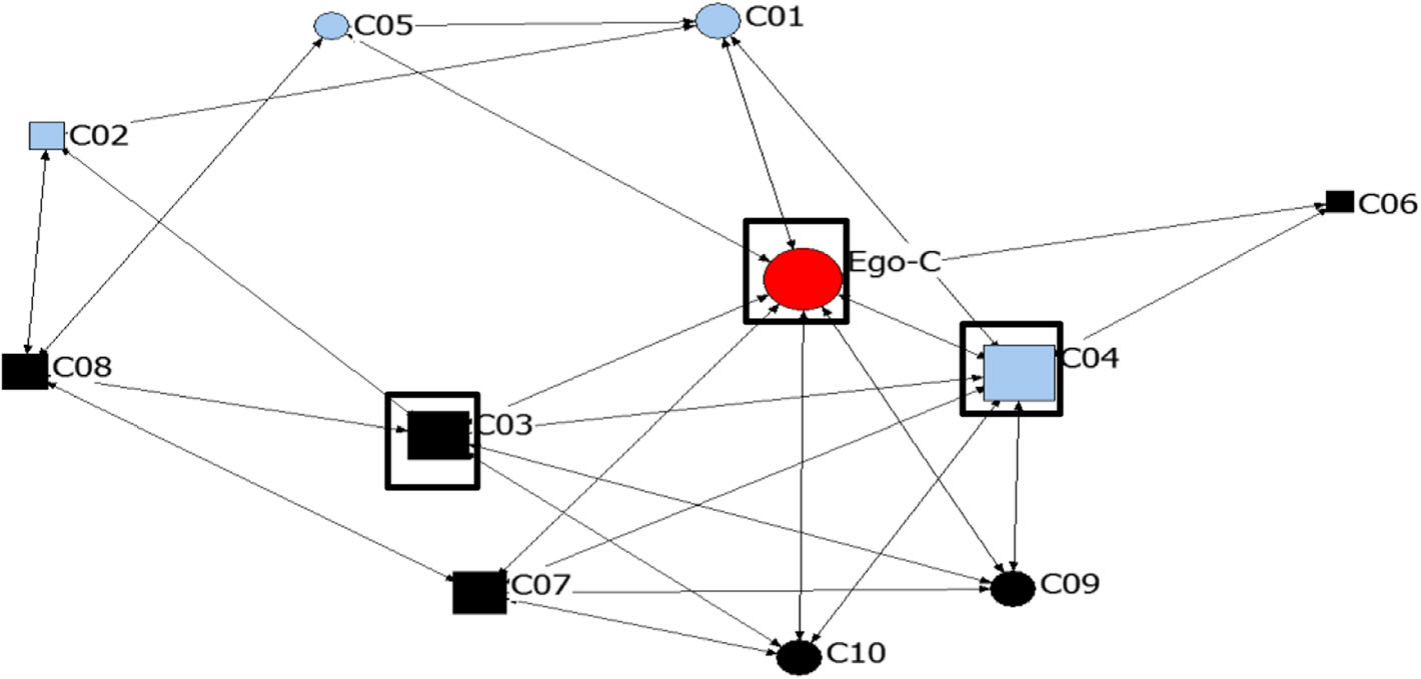
Network map for SNA-C – the least dense (0.46) ego network

**Fig. 2:**
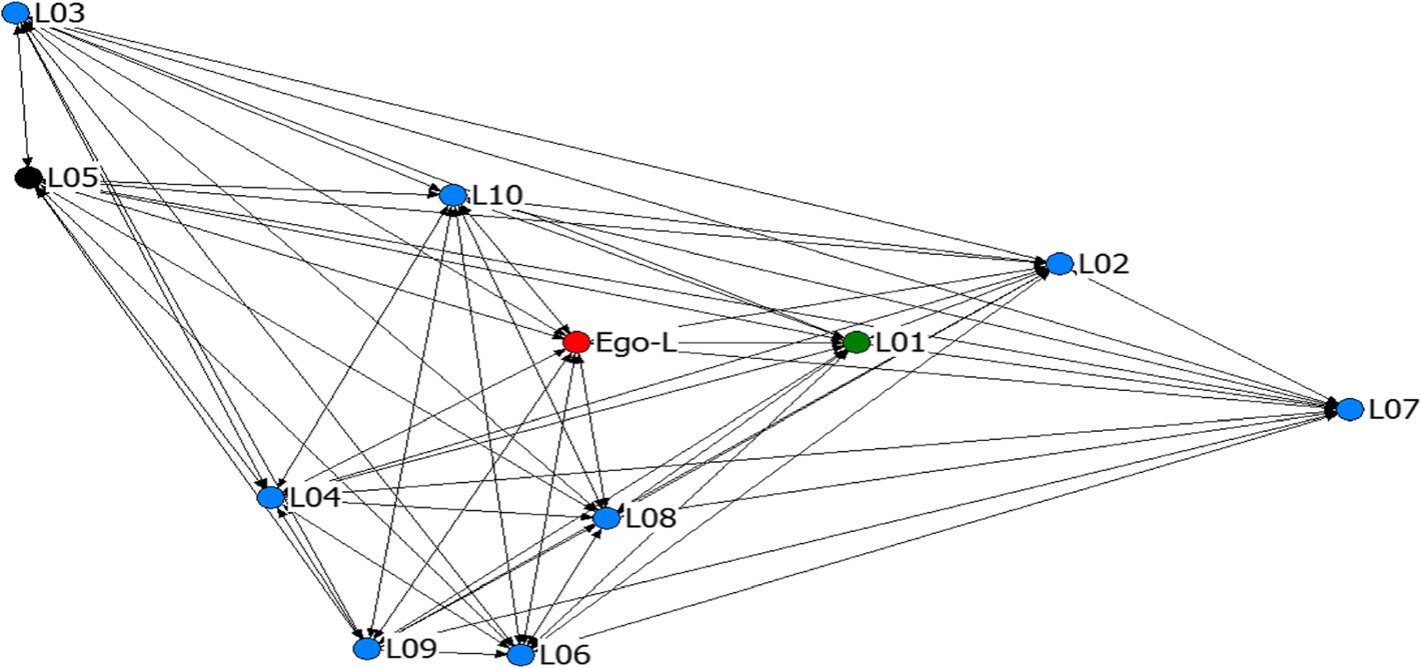
Network map for SNA-L showing a perfectly dense ego network

The networks in this study were generally dense, with density ranging from 0.46 to 1 (see Figs. 1 and 2; Key to node coding on the sociograms is presented in Table 4). Nine of 10 networks in site A and all networks in Site B had densities > 0.5 while 3 networks have 100% density, meaning that all actors in the network talk to each other about injectable contraceptives. Also, there was low network centralization in most networks, ranging from 0 to 73.4%. However, density in most networks is greater than 0.5 (see Table 5). This suggests that as density (connection among actors) increases, the tendency for power or prominence to be concentrated in a few actors reduces.

**Table 4:** Key to node coding on sociograms

Variable	Code	Label
Shape	Circle	Users of injectable contraceptives
	Square	Non-users of injectable contraceptives
Colour	Red	Ego
	Pink	Relatives and close friends
	Blue	Close friends
	Black	Co-worker
	Green	Neighbour
Size of node	Proportional to the degree centrality of actor	
Boxes around nodes^a^	Actors with the top three highest betweeness centrality values	

^a^Sociograms without boxes are those where all the actors have equal betweeness centrality values

**Table 5:** Network density and degree centrality

Network	No. of ties present^a^	Density	Ego’s degree centrality	Average degree centrality	Ego nBetweeness	Network Average nBetweeness	CBD injectable contraceptive use
SITE A (GOMBE)							
SNA-A	84	0.76	9	7.64	4.37	2.45	8
SNA-B	96	0.87	10	8.73	2.14	1.34	10
SNA-C	50	0.46	8	4.55	23.07	4.80	4
SNA-D	94	0.86	7	8.55	1.56	1.62	7
SNA-E	108	0.98	10	9.82	0.25	0.20	10
SNA-F	88	0.98	5	8.00	4.58	1.99	7
SNA-G	86	0.78	7	7.82	2.56	2.41	5
SNA-H	92	0.84	8	8.37	1.60	2.29	8
SNA-I	92	0.84	8	8.36	1.15	1.67	9
SNA-J	74	0.67	7	6.73	3.11	2.80	8
SITE B (YEMALTU-DEBA)							
SNA-K	74	0.67	10	6.73	16.41	2.36	10
SNA-L	110	1.00	10	10.00	0	0	10
SNA-M	110	1.00	10	10.00	0	0	9
SNA-N	76	0.69	10	6.91	9.78	2.80	10
SNA-O	82	0.75	10	7.45	10.63	2.05	10
SNA-P	56	0.51	10	5.09	30.56	2.95	10
SNA-Q	56	0.51	2	5.09	0	2.89	3
SNA-R	110	1.00	10	10.00	0	0	3
SNA-S	96	0.87	10	8.73	2.64	1.30	10
SNA-T	80	0.73	10	7.27	8.52	2.48	9

^a^Number of possible ties = 110

In networks with 100% density, injectable contraceptive use was either very high or very low. In four out of the 20 networks (D, H, I, Q), the egos were less prominent than the alters in the network (Table 5).

#### Degree centrality and betweeness centrality among actors

**Fig. 3:**
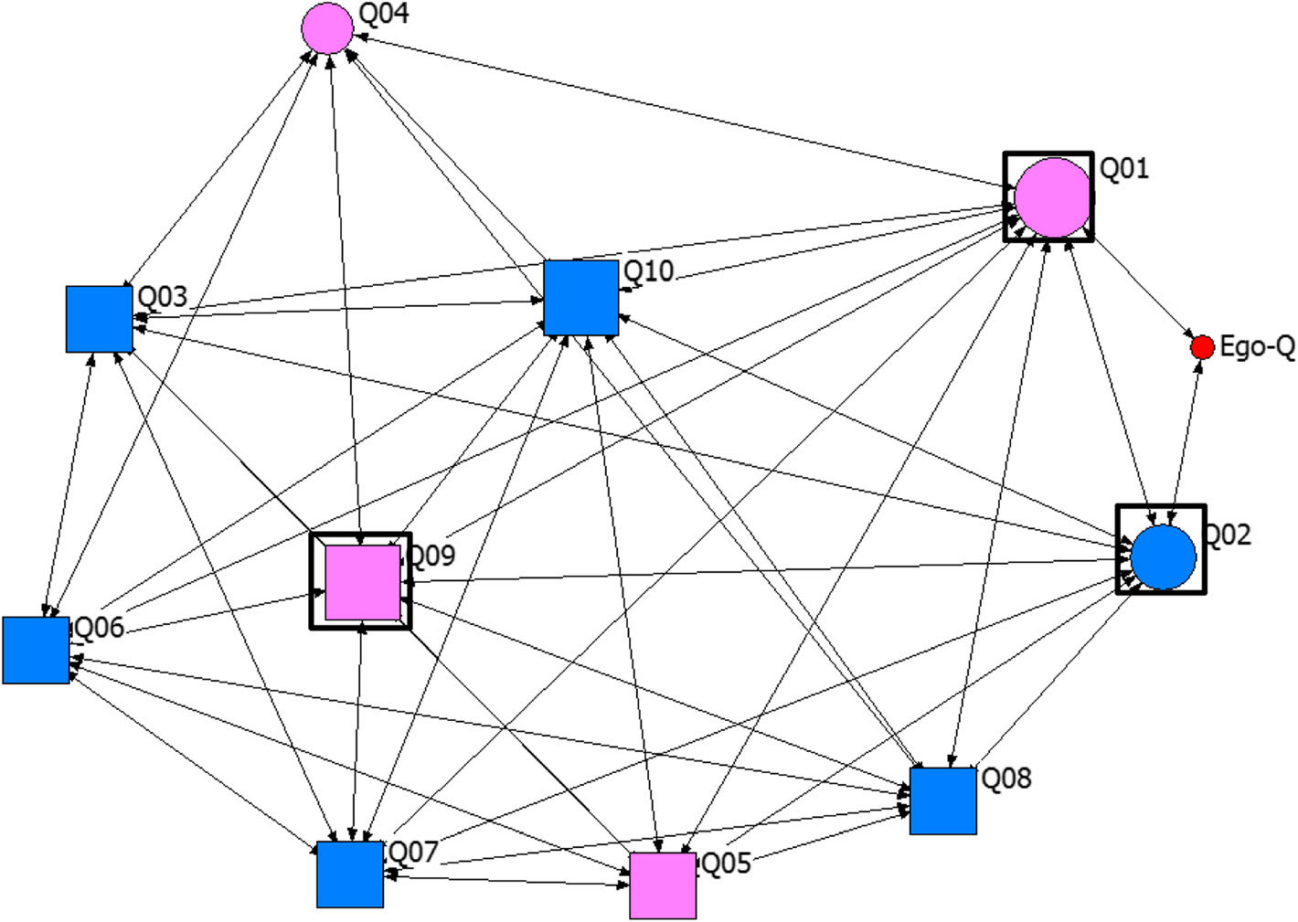
Network map for SNA-Q – showing a peripheral ego with low degree centrality

**Fig. 4:**
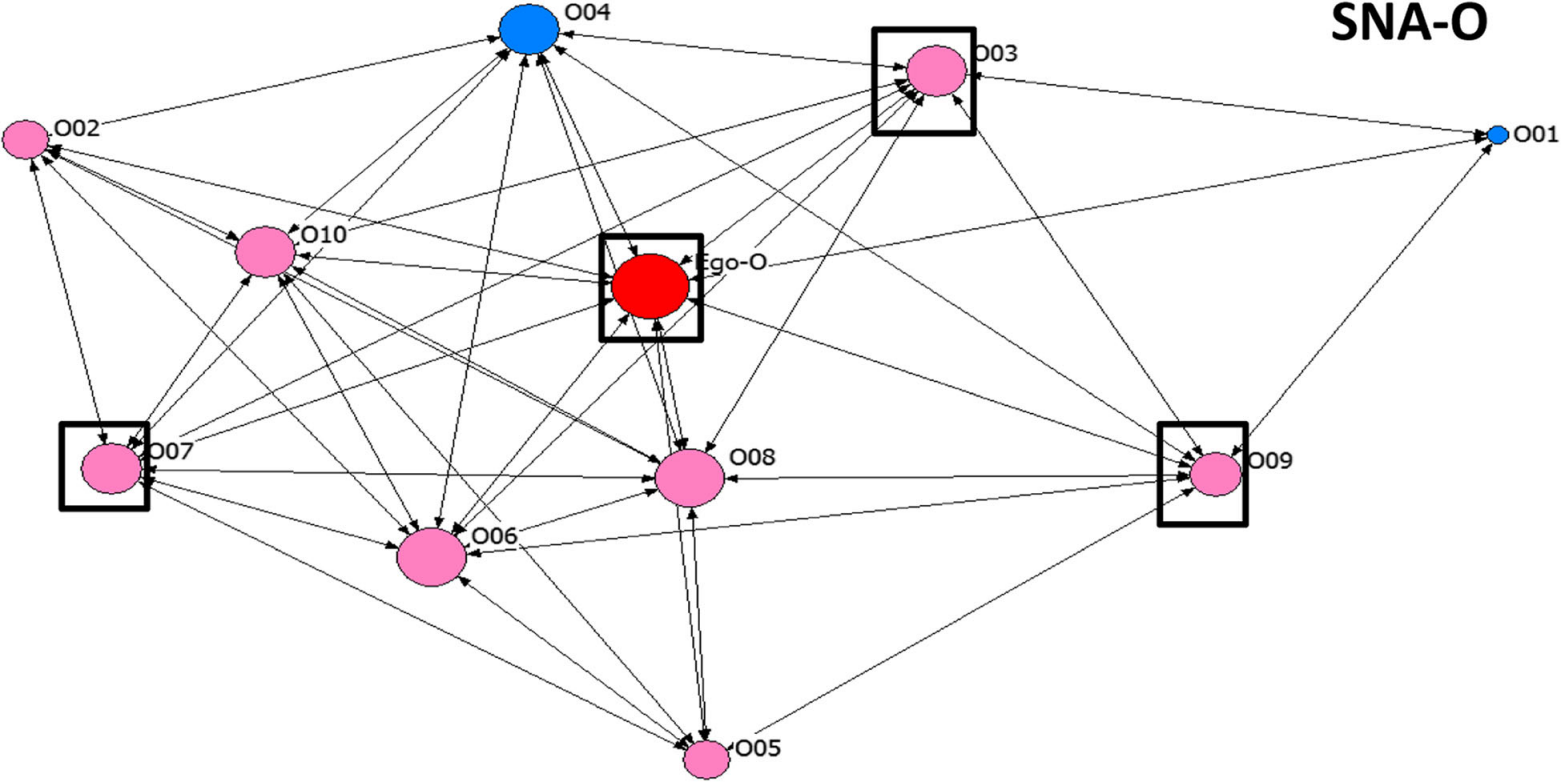
Network map for SNA-O showing a peripheral actor

In 18 of the networks, egos had very high degree centrality (7 or higher). In site B, almost all egos (9 out of 10) had degree centrality of 10 (maximum). In Fig. 2, the ego has a degree centrality of 10 while Fig. 3 shows an example of a sociogram where ego has low degree centrality (peripheral). In about one-third of the networks (6 out of 20), the egos had lower degree centrality with fewer ties compared with the average degree centrality of alters in the networks (see SNA-D, SNA-F, SNA-G, SNA-H, SNA-I and SNA-Q in Table 6). The majority of the actors with high degree centrality also had high betweeness centrality. Also, a greater proportion of actors with high betweeness centrality were users of CBD injectable contraceptives (43 out of 60). Although, in a few of the networks, some of the actors with the highest betweeness were not users of injectable contraceptives (see Fig. 1 above). About half of the egos also have high betweeness centrality (11 out of 20). Similarly, most of the alters with high betweeness centrality were egos’ neighbors, followed by their relatives and close friends (See Figs. 1, 3 and 4). Generally, the majority of actors in these networks used injectable contraceptives (Table 3).

**Table 6:** Group level E-I indices^a^

Network	Ethnicity		Age of actors		Education		Marriage type	
	Hausa	Others	< 30	≥30	Less than secondary	At least secondary	Monogamous	Polygamous
SITE A (GOMBE)								
SNA-A	0.333	0.000	−0.106	0.135	0.179	0.022	−0.460	0.619
SNA-B	−1.000	–	0.750	−0.650	1.000	−0.793	0.256	0.019
SNA-C	−0.674	1.000	1.000	−0.826	0.111	−0.375	0.091	−0.143
SNA-D	−1.000	–	0.556	− 0.373	0.500	−0.226	0.163	− 0.020
SNA-E	−1.000	–	1.000	−0.796	− 0.333	− 0.370	− 0.017	0.184
SNA-F	−1.000	–	1.000	− 0.772	0.778	− 0.543	0.273	−0.227
SNA-G	−0.795	1.000	0.778	−0.529	0.368	0.083	0.714	−0.667
SNA-H	−0.534	0.789	–	−1.000	–	− 1.000	0.538	−0.394
SNA-I	−1.000	–	1.000	−0.810	–	−1.000	0.789	−0.534
SNA-J	0.429	−0.130	–	−1.000	1.000	−0.688	0.143	−0.304
SITE B (YEMALTU-DEBA)								
SNA-K	0.478	−0.333	0.714	−0.600	−1.000	–	−0.073	0.152
SNA-L	0.400	−0.200	0.400	−0.200	−0.600	0.800	−0.600	0.800
SNA-M	0.600	−0.400	0.400	−0.200	−0.600	0.800	0.600	−0.400
SNA-N	−1.000	–	−0.333	0.143	0.273	−0.023	−0.167	0.429
SNA-O	−1.000	–	−0.377	0.810	0.667	−0.310	0.077	−0.023
SNA-P	−1.000	–	−1.000	–	0.200	−0.333	−0.500	0.250
SNA-Q	−0.154	0.375	−0.722	0.667	0.314	−0.061	−0.722	0.667
SNA-R	−0.200	0.400	−0.600	0.800	0.400	−0.200	−0.600	0.800
SNA-S	−1.000	–	−0.429	0.538	0.163	−0.057	−0.057	0.163
SNA-T	−1.000	–	−0.400	0.800	−0.333	0.652	−0.746	1.000

^a^E-I index is the number of ties external to the groups minus the number of ties that are internal to the group divided by the total number of ties. This value can range from 1 to −1

### Communication about community-based distributed injectable contraceptives

In both sites A and B, egos reported that alters discuss family planning issues often (95 and 97% respectively). Table 6 shows that in 15 of the 20 networks across the two sites, actors who were Hausas tended to talk about the community-based distributed injectable method more with fellow Hausas than with women of other ethnic affiliations. Furthermore, the networks showed homophily with respect to age – participants who were 30 years or older tended to talk more with women in their age group – although a look through the lens of location shows that the homophily was more pronounced in Gombe LGA (9 of 10 networks) than in Yemaltu-Deba LGA (3 of 10 networks). In addition, a tendency to communicate among groups with similar characteristics was also observed among actors who had at least secondary school education. Although marriage type is a factor of homophily, actors in polygamous relationships were more homophilic in Gombe whereas, in Yemaltu-Deba, it was actors in monogamous marriages who had homophilic communication interactions.

Majority of actors in the networks showed homophily according to ethnicity (88.6%), injectable contraceptive use (81.4%) and age (80.0%). This suggests that actors in this study interact more based on their ethnic affiliations as well as their use of injectable contraceptives and age. Actors demonstrated the least homophily according to marriage type (49.5%). Table 7 shows details of homophily among women in the study.

**Table 7:** Sources of homophily among actors according to specific characteristics (*N* = 220)

Variable	Egos (*N* = 20)		Alters (*N* = 200)		Total	
	n	%	n	%	n	%
Ethnicity	20	100.0	175	87.5	195	88.6
Injectable contraceptive use	17	85.0	162	81.0	179	81.4
Age	18	90.0	158	79.0	176	80.0
Education	13	65.0	133	66.5	146	66.4
Marriage type	11	55.0	98	49.0	109	49.5

In some of the networks, there were peripheral actors who had very few ties with other actors in the network (see Fig. 4). In addition to having low degree centrality, these actors also had low betweeness centrality.

## Discussion

This study revealed that most of the networks have high densities. Also, actors with high degree centrality showed prominence in their networks. In addition, common sources of homophily among actors in networks were ethnicity, age, education, and marriage type. The implications of these findings are discussed below.

According to Haythornthwaite, actors in dense networks communicate more amongst themselves than they do in loose networks [39]. Since dense networks make for easier communication and organization of activities among actors [27, 40], it would seem to be easier for the knowledge and use of injectable contraceptives to spread among these networks. This is probably due to a higher degree of trust and support among actors in the network, given that more than half of the alters were family or close friends [40].

However, if a number of actors in a network are non-users of injectable contraceptives, it might be difficult for these actors to adopt the innovation in a dense network since individual decisions are largely swayed by the common opinion in the network [27, 41]. According to Prell, [40] actors in a dense network may embrace incorrect information and could be less open to new information thus limiting other players in the network, and ultimately restricting the dissemination of accurate information about health innovations. This assertion is buttressed by the findings of this study which showed that networks with the highest densities may have either a very high *or* very low proportion of injectable contraceptive users, suggesting that actors inspire one another in the decision to use or not to use injectable contraceptive since “everyone knows everyone’s business” [27]. Thus, it is imperative to get the right message about injectable contraceptives across to potential users of this innovation in these closely-knit communities.

In dense networks like in this study, each actor relates with every other actor in the network and no particular person is prominent [40]. Thus, the use of injectable contraceptives is more likely due to group influence than individual decisions by members of the network [27]. Also, in a very dense network, any of the actors in the network could easily become a leader championing the spread of the innovation within the network [3, 27]. Thus, the network is robust and resilient and not dependent on few key players [40]. Conversely, any one of the players could also be a source of false information about injectable contraceptives, thereby slowing down its adoption and use in the network. This means that in networks with high densities, there are no information “gatekeepers” that other actors can rely on to get connected to the network [27]. Likewise, about a quarter of the egos were not prominent in their networks. Consequently, the egos might not necessarily be the most popular or most active in the spread of information about injectable contraceptive use since most of the networks are resilient and not centralized around a few actors [40]. Also, some of the alters might be early adopters themselves.

While it is not possible to refer with certainty to peripheral actors as links to other networks, because the study focused on ego networks, nevertheless, these outliers may have links to other networks. This so-called “strength of weak ties” [42], suggests that the peripheral actors could be formidable players in the diffusion process by serving as conduits through which networks associated with them get pertinent information from the environment.

Furthermore, our study revealed that actors with high degree centrality tend to also have high betweeness centrality. This suggests that actors with high degree centrality were relatively more prominent in the network and able to control the flow of information about injectable contraceptives [27, 28]. Since most actors in this study with high betweeness centrality were injectable contraceptive users, this may have a positive ripple effect on the spread of the innovation in these communities. Actors with high betweeness centrality might be useful as peer educators since they are able to act as ‘middle men’ between other actors in their networks and possibly link their network to other groups [43]. Since the majority of the actors with high betweeness centrality in this study were users of community-based distributed injectable contraceptives, they may also act as “champions” and opinion leaders in their communities [3]. These actors might be able to play this role because they usually control the flow of information in the network due to their ties to several other actors; thus they are able to organize and spread information about injectable contraceptives to the whole network [40].

In addition, few actors who have high betweeness centrality in some networks in the study were not users of injectable contraceptives. This may have implications for the quality of information shared in these networks about injectable contraceptives [27]. It may also mean that actors who perhaps control information about injectable contraceptives were not necessarily users themselves. This may have a negative influence on the adoption and use of injectable contraceptives in such types of networks.

Moreover, most of the networks in this study were homophilic with respect to ethnicity, age, education, and marriage type. Although, the homophily due to ethnicity observed in this study could be because Hausa is the major ethnic group in the research area. Ethnicity has been described as the greatest source of homophily in social networks followed by age, religion, education, occupation and, gender [7]. Ethnicity has also been considered as an important determinant of social group membership [7, 44]. Homophily from race and ethnicity has been reported to permeate marriage, work, friendship, acquaintanceship, to those in whom individuals confide or discuss important matters with before making their decisions [7, 45]. Thus, working through natural groupings may help to facilitate the diffusion of information and better uptake in the process of scaling up of community-based injectable contraceptives in Gombe and similar communities. At the same time, given that conflict in Gombe State might also be sustained through networks that promote religious and sometimes ethnic homophily, the use of such networks to support the scale up of community-based distributed injectable contraceptives requires political sensitivity and care.

In this study, actors preferred to interact and share information about injectable contraceptives within their age groups. Homophily resulting from age homogeneity has been reported to be long-lasting and usually very strong, probably because these ties often start from childhood [7, 46]. In addition, just as it was found in this study, education and occupation (which are principal determinants of social class in many contexts, including Gombe), have been shown to demonstrate strong homophily in social networks [7]. Education, in particular has been reported to influence the uptake of health innovations [47, 48]. Thus, these characteristics – age, education, and occupation – should be considered when engaging peer educators in the diffusion of innovations like the community-based injectable contraceptives. However, one drawback of homophilic networks is that it is easier for the network to be contaminated with inaccurate or false information or myths about injectable contraceptives, since the actors in the networks have related sources of knowledge [40].

Nevertheless, this study is limited by the subjectivity of egos as information obtained was solely from the egos’ points of view. Still, it is this very subjectivity which also provides valuable data and enables a better understanding of the influence of network phenomenon – themselves subjective - on uptake and diffusion of health innovations. In addition, almost all alters and egos in this study were married thereby limiting the scope of the application of these findings.

## Conclusion

This study shows the application and feasibility of ego social network analysis in identifying and disseminating health innovation through natural groupings in the community. It also illustrates how communication and social interactions among women of reproductive age might influence the uptake and diffusion of community-based injectable contraceptives by others. Additionally, this work shows how the exploitation of network phenomena in homophilic and dense networks may have positive health externality such as passive diffusion of health innovations past the point of introduction [49]. The interrelatedness of network members’ decision to adopt a health innovation was also illustrated by the findings of this study.

Thus, it is recommended that health messages about the community-based distribution of injectable contraceptives be carefully considered for accuracy and appropriateness, before being disseminated in these closely-knit communities. Also, highly connected or prominent individuals should be identified in the communities to serve as peer educators or “champions” in the scale up process. These champions should be identified within different groups for example marital, ethnic, age, educational, and employment groups, since community members tend to interact more according to these groupings.

## Supplementary information

**Additional file 1:** Research tools.

## Data Availability

The data used and/or analyzed in this study are available from Dr. Oluwaseun Akinyemi on request.

## Abbreviations

CBD: Community-based distribution
CHEWs: Community health extension workers
CHWDIC: Community health worker-delivered injectable contraceptives
CPR: Contraceptive prevalence rates
LGA: Local Government Area
LMICs: Low- and middle-income countries
NGO: Non-governmental organization
SNA: Social network analysis

## References

[1] Subramanian S, Naimoli J, Matsubayashi T, Peters DH (2011). Do we have the right models for scaling up health services to achieve the millennium development goals?. BMC Health Serv Res.

[2] Manham L, Hanson K (2010). Scaling up in international health: what are the key issues? Review. Health Policy Plan.

[3] Valente TW (2012). Network interventions. Science.

[4] Valente TW, Ritt-Olson A, Stacy A, Unger JB, Okamoto J, Sussman S (2007). Peer acceleration: effects of a social network tailored substance abuse prevention program among high-risk adolescents. Addiction.

[5] Scott J (2017). Social network analysis: Sage.

[6] Wonodi C, Privor-Dumm L, Aina M, Pate A, Reis R, Gadhoke P, Levine O (2012). Using social network analysis to examine the decision-making process on new vaccine introduction in Nigeria. Health Policy Plan.

[7] McPherson M, Smith-Lovin L, Cook JM (2001). Birds of a feather: Homophily in social networks. Annu Rev Sociol.

[8] OlaOlorun FM, Hindin MJ (2014). Having a say matters: influence of decision-making power on contraceptive use among Nigerian women ages 35–49 years. PLoS One.

[9] Adekunle AO, Otolorin EO (2000). Evaluation of the Nigerian population policy--myth or reality?. Afr J Med Med Sci.

[10] National Population Commission (2008). Nigeria demographic and health survey 2008. Nigeria and ICF Macro.

[11] Factsheet on the world malaria report 2013. http://www.who.int/malaria/media/world_malaria_report_2013/en/. Accessed 15 July 2018.

[12] World Health Organization (2014). World health statistics 2014.

[13] National Population Commission, ICF International (2014). Nigeria demographic and health survey 2013. Abuja, Nigeria.

[14] FHI (2010). The effectiveness of community-based access to injectable contraceptives in Nigeria: a technical report.

[15] Conway MD, Rizzuto CD, Weiss LM (2008). A better way to speed the adoption of vaccines. McKinsey Quarterly.

[16] Bradley E, Curry L, Pérez-Escamilla R, Berg D, Bledsoe S, Ciccone D (2011). Dissemination, diffusion and scale up of family health innovations in low-income countries. Yale Global Health Leadership Institute.

[17] Malarcher S, Meirik O, Lebetkin E, Shah I, Spieler J, Stanback J (2011). Provision of DMPA by community health workers: what the evidence shows. Contraception.

[18] Stanback J, Spieler J, Shah I, Finger WR (2010). Community-based health workers can safely and effectively administer injectable contraceptives: conclusions from a technical consultation. Contraception.

[19] Hoke T, Brunie A, Krueger K, Dreisbach C, Akol A, Rabenja NL, Olawo A, Stanback J (2012). Community-based distribution of injectable contraceptives: introduction strategies in four sub-Saharan African countries. Int Perspect Sex Reprod Health.

[20] Prata N, Gessessew A, Cartwright A, Fraser A (2011). Provision of injectable contraceptives in Ethiopia through community-based reproductive health agents. Bull World Health Organ.

[21] USAID: Community-Based Access to Injectable Contraception Educational Tour Guidance Package. In Community-Based Access to Injectable Contraceptives Toolkit (2011). Available from: https://toolkits.knowledgesuccess.org/sites/default/files/1-Introduction_FINAL_Branded.pdf. Accessed 2 July 2018.

[22] Levine R (2004). Millions saved: proven successes in global health: Peterson Institute.

[23] Brunie A, Hoke TH, Razafindravony B (2011). Community-based distribution of injectable contraceptives in an African setting: community trial in Madagascar. Sante (Montrouge, France).

[24] Eaton J, McCay L, Semrau M, Chatterjee S, Baingana F, Araya R, Ntulo C, Thornicroft G, Saxena S (2011). Scale up of services for mental health in low-income and middle-income countries. Lancet.

[25] Kawonga M, Blaauw D, Fonn S (2015). Exploring the use of social network analysis to measure communication between disease programme and district managers at sub-national level in South Africa. Soc Sci Med.

[26] Kawonga M (2017). Applying social network analysis to explore the extent of communication interactions between HIV program and general health service managers in South Africa. In. London.

[27] Hawe P, Webster C, Shiell A (2004). A glossary of terms for navigating the field of social network analysis. J Epidemiol Community Health.

[28] Marks J, Barnett LM, Foulkes C, Hawe P, Allender S (2013). Using social network analysis to identify key child care center staff for obesity prevention interventions: a pilot study. J Obes.

[29] Bradley EH, Curry LA, Taylor LA, Pallas SW, Talbert-Slagle K, Yuan C, Fox A, Minhas D, Ciccone DK, Berg D (2012). A model for scale up of family health innovations in low-income and middle-income settings: a mixed methods study. BMJ Open.

[30] Curry L, Taylor L, Pallas SW, Cherlin E, Pérez-Escamilla R, Bradley EH (2013). Scaling up depot medroxyprogesterone acetate (DMPA): a systematic literature review illustrating the AIDED model. Reprod Health.

[31] Valente TW (2010). Social networks and health: models, methods, and applications: Oxford University press.

[32] Rogers EM (2002). Diffusion of preventive innovations. Addict Behav.

[33] Gombe State Ministry of Health (2012). Gombe state framework for the implementation of expanded access to family planning services.

[34] Gombe State. http://www.nigeria.gov.ng/index.php/2016-04-06-08-39-54/north-east/gombe-state. Accessed 15 July 2018.

[35] BBC (2015). Boko Haram attack Gombe state capital. Africa – News and Analysis. vol. 2015: Africa Journalism.

[36] Maina M (2015). Boko Haram attacks Kasheri in Gombe. Daily post vol 2015.

[37] NPC and ICF International (2014). Nigeria demographic and health survey In. Nigeria, and Rockville, Maryland, USA.

[38] Increasing access to contraceptives in Nigeria through task-shifting to Community Health Extension Workers (CHEWS). http://arfh-ng.org/increasing-access-contraceptives-nigeria-task-shifting-community-health-extension-workers-chews/. Accessed 15 July 2018.

[39] Haythornthwaite C (1996). Social network analysis: an approach and technique for the study of information exchange. Libr Inf Sci Res.

[40] Prell C, Hubacek K, Reed M (2009). Stakeholder analysis and social network analysis in natural resource management. Soc Nat Resour.

[41] Bott E (1957). Family and social network: roles.

[42] Granovetter M (2003). The strength of weak ties. Networks in the knowledge economy.

[43] Otte E, Rousseau R (2002). Social network analysis: a powerful strategy, also for the information sciences. J Inf Sci.

[44] Ellison NB (2007). Social network sites: definition, history, and scholarship. J Comput-Mediat Commun.

[45] South SJ, Felson RB (1990). The racial patterning of rape. Soc Forces.

[46] Fischer CS (1982). To dwell among friends: personal networks in town and city: University of Chicago press.

[47] Sabates R, Feinstein L (2006). The role of education in the uptake of preventative health care: the case of cervical screening in Britain. Soc Sci Med.

[48] Atun RA, Menabde N, Saluvere K, Jesse M, Habicht J (2006). Introducing a complex health innovation—primary health care reforms in Estonia (multimethods evaluation). Health Policy.

[49] Smith KP, Christakis NA (2008). Social networks and health. Annu Rev Sociol.

