# Supplementary material for: Innovation diffusion: how homogenous networks influence the uptake of community-based injectable contraceptives
Source: BMC Public Health. 2019 Nov 14;19:1520. doi: 10.1186/s12889-019-7819-5 (PMC6857216; doi:10.1186/s12889-019-7819-5)
Supplement: Supplementary file 1 — Additional file 1: Research tools. [file 12889_2019_7819_MOESM1_ESM.docx]

## **SNA questionnaire: Interviews with women who have adopted injectable contraceptives delivered by CHEWs**

**Project Title: Scale up of community-based injectable contraceptives in Gombe State, Nigeria**

| Participant code |  |
| --- | --- |

| Date of interview: | DD | MM | YYYY |
| --- | --- | --- | --- |

| Place of residence |  |
| --- | --- |
| State |  |
| Local municipality |  |
| Town |  |
| Village / suburb |  |

1. **Participant (ego) attributes**
2. Age at last birthday (in years) ______________
3. Highest level of education completed
4. No formal education
5. Arabic school
6. Primary school
7. Secondary school
8. Tertiary education (please specify type: _______________________)
9. Occupation _______________________________
10. Marital status
11. Single
12. Cohabiting
13. Married
14. Separated
15. Divorced
16. Widowed
17. Other (please specify)_______________________
18. Type of marriage
19. Monogamy
20. Polygamy
21. Others (please specify)______________________
22. Ethnicity
23. Hausa
24. Ibo
25. Yoruba
26. Others (please specify)____________________
27. When did you start using injectable contraceptives provided by a CHEW?

Year _________ month ______

1. Where / how do you receive the injectable contraceptives from a CHEW?
2. At home
3. At the market
4. At the village square
5. Other (please specify)______________________________
6. **Name generation and ego-alter relationship**
7. Can you please tell me who are the women that you interact with regularly (e.g. those you talk to, share activities with)? Please mention only women who are of child-bearing age.
   1. Please just tell me their first names or initials.

**Insert responses on the data capture sheet**

- 1. For each person you have mentioned, I would like to know what is your relationship with her :
     1. Relative / family member
     2. Close friend
     3. Co-worker
     4. Neighbor
     5. Someone you meet at church/mosque
     6. Someone you meet at the market / place of trade
     7. Other type of relationship (please specify) _________________?

**Insert coded responses on the data capture sheet**

1. Kindly give me some information about each of the persons you mentioned earlier.

**Insert coded responses on the data capture sheet**

1. How long have you known her (years and months) ____________________
2. Roughly how old is she? (Age in years) ______________
3. What is her highest level of education?
4. No formal education
5. Arabic school
6. Primary school
7. Secondary school
8. Tertiary education (please specify type: _______________________)
9. What is her occupation? _______________________________
10. What is her marital status? Is she:
11. Single
12. Cohabiting
13. Married
14. Separated
15. Divorced
16. Widowed
17. Other (please specify)_______________________
18. What Type of marriage does she have?
19. Monogamy
20. Polygamy
21. Others (please specify______________________)
22. What is her Ethnicity? Is she:
23. Hausa
24. Ibo
25. Yoruba
26. Others (please specify)____________________
27. To the best of your knowledge, does xx use contraceptives / family plan?

*1=yes*

*2=no*

*3. Don’t know*

1. To the best of your knowledge, does xx use injectable contraceptives provided by a CHEW?

*1=yes*

*2=no*

*3. Don’t know*

**C. Ego-alter discussions on CBD of contraceptives**

I. From time to time, people discuss important personal matters with other people. Looking back over the last six months:

1. How frequently have you discussed any issues about family planning with xx?
   - 1. *Never*
     2. *Very often;*
     3. *Occasionally*
     4. *Rarely*
2. If “Never”: is there any reason why you do not discuss issues about family planning with xx?

**Narrative (capture open-ended responses)**

_________________________________________________________

_________________________________________________________

III. Let us now focus specifically on the women you said you have spoken to about family planning in the last 6 months. For each woman I would like you to answer the following questions

1. Since the time that you yourself started using injectable contraceptives provided by a CHEW, have you ever shared with her your experience of receiving injectable contraceptives from a CHEW??

*1=yes*

*2=no*

- If “No”: Is there a reason why you have not talked with her about your experience of using injectable contraceptives from a CHEW?

**Narrative (capture open-ended responses)**

_________________________________________________________

_________________________________________________________

b. Have you ever specifically recommended to xx to also start using CBD injectable contraceptives from a CHEW?

*1=yes*

*2=no*

- If “No”: How likely are you to recommend CBD injectable contraceptive to xx?

*1=very likely;*

*2=somewhat likely;*

*3=very unlikely*

**D. Alter-alter relationship**

1. To the best of your knowledge, what is the relationship between each of the women you listed above? Would you say xx and xx are:
   - - 1. Strangers (don’t know each other)
       2. Family/kinship
       3. Close friends
       4. Co-workers
       5. Neighbours
       6. Meet each other at church/mosque
       7. Meet each other at the market / place of trade
       8. Share other type of relationship (please specify) _________________?

**Code the response in the relevant cell in the SNA Matrix below (the clear cells only)**

| **Persons** |  |  |  |  |  |  |  |  |  |  |
| --- | --- | --- | --- | --- | --- | --- | --- | --- | --- | --- |
|  |  |  |  |  |  |  |  |  |  |  |
|  |  |  |  |  |  |  |  |  |  |  |
|  |  |  |  |  |  |  |  |  |  |  |
|  |  |  |  |  |  |  |  |  |  |  |
|  |  |  |  |  |  |  |  |  |  |  |
|  |  |  |  |  |  |  |  |  |  |  |
|  |  |  |  |  |  |  |  |  |  |  |
|  |  |  |  |  |  |  |  |  |  |  |
|  |  |  |  |  |  |  |  |  |  |  |
|  |  |  |  |  |  |  |  |  |  |  |

1. In your opinion, what is the likelihood that the women you listed above will talk among themselves about or recommend to one another using the injectable contraceptive distributed in the community by CHEWs?
2. Never
3. Very likely
4. Somewhat likely
5. Unlikely

| **Persons** |  |  |  |  |  |  |  |  |  |  |
| --- | --- | --- | --- | --- | --- | --- | --- | --- | --- | --- |
|  |  |  |  |  |  |  |  |  |  |  |
|  |  |  |  |  |  |  |  |  |  |  |
|  |  |  |  |  |  |  |  |  |  |  |
|  |  |  |  |  |  |  |  |  |  |  |
|  |  |  |  |  |  |  |  |  |  |  |
|  |  |  |  |  |  |  |  |  |  |  |
|  |  |  |  |  |  |  |  |  |  |  |
|  |  |  |  |  |  |  |  |  |  |  |
|  |  |  |  |  |  |  |  |  |  |  |
|  |  |  |  |  |  |  |  |  |  |  |

**THANK YOU FOR YOUR TIME**
